# Treatment Efficacy Analysis in Acute Ischemic Stroke Patients Using In Silico Modeling Based on Machine Learning: A Proof-of-Principle

**DOI:** 10.3390/biomedicines9101357

**Published:** 2021-09-29

**Authors:** Anthony Winder, Matthias Wilms, Jens Fiehler, Nils D. Forkert

**Affiliations:** 1 Department of Radiology, University of Calgary, Calgary, AB T2N 2T9, Canada; matthias.wilms@ucalgary.ca (M.W.); nils.forkert@ucalgary.ca (N.D.F.); 2 Hotchkiss Brain Institute, University of Calgary, Calgary, AB T2N 4N1, Canada; 3 Department of Diagnostic and Interventional Neuroradiology, University Medical Center Hamburg-Eppendorf, 20251 Hamburg, Germany; fiehler@uke.de; 4 Department of Clinical Neuroscience, University of Calgary, Calgary, AB T2N 2T9, Canada; 5 Alberta Children’s Hospital Research Institute, University of Calgary, Calgary, AB T3B 6A8, Canada

**Keywords:** stroke, brain ischemia, thrombectomy, machine learning, predictive modeling, precision medicine, tissue outcome prediction, efficacy analysis

## Abstract

Interventional neuroradiology is characterized by engineering- and experience-driven device development with design improvements every few months. However, clinical validation of these new devices requires lengthy and expensive randomized controlled trials. This contribution proposes a machine learning-based in silico study design to evaluate new devices more quickly with a small sample size. Acute diffusion- and perfusion-weighted MRI, segmented one-week follow-up imaging, and clinical variables were available for 90 acute ischemic stroke patients. Three treatment option-specific random forest models were trained to predict the one-week follow-up lesion segmentation for (1) patients successfully recanalized using intra-arterial mechanical thrombectomy, (2) patients successfully recanalized using intravenous thrombolysis, and (3) non-recanalizing patients as an analogue for conservative treatment for each patient in the sample, independent of the true group membership. A repeated-measures analysis of the three predicted follow-up lesions for each patient revealed significantly larger lesions for the non-recanalizing group compared to the successful intravenous thrombolysis treatment group, which in turn showed significantly larger lesions compared to the successful mechanical thrombectomy treatment group (*p* < 0.001). A groupwise comparison of the true follow-up lesions for the three treatment options showed the same trend but did not reach statistical significance (*p* = 0.19). We conclude that the proposed machine learning-based in silico trial design leads to clinically feasible results and can support new efficacy studies by providing additional power and potential early intermediate results.

## 1. Introduction

Stroke is a major cause of death and disability in the world. For example, the annual cost for stroke patients amounts to approximately CAD 2.8 billion in the Canadian health care system [1], USD 28 billion for the US health care system [2], and EUR 60 billion in the European health care systems [3]. Even a small improvement in clinical stroke outcomes could improve the daily life of many patients and result in significant cost savings in the health care system. The majority of all strokes are caused by an artery blockage due to a blood clot, the so-called ischemic stroke [4]. Such a blockage leads to a partial or complete restriction of blood flow to the brain tissue supplied by this artery and may result in irreversible damage to the brain cells in that region.

For many years, intravenous tissue plasminogen activator (IV tPA) therapy was the only acute ischemic stroke patient treatment together with stroke unit care. However, IV tPA is only effective in 30–40% of treated patients to achieve early recanalization [5]. Multiple randomized controlled trials have shown an overwhelming efficacy of intra-arterial therapy for treatment of large vessel occlusions with considerably higher recanalization rates compared to IV tPA alone [6]. Based on the results of these recent randomized controlled trials, new medical devices for intra-arterial therapy are currently being developed with the aim to increase recanalization rates. However, the actual clinical benefit of new devices must be shown in randomized control trials with large patient samples. For example, 500 patients (233 intra-arterial therapy, 267 IV tPA controls) were recruited in the MR CLEAN study [7], 315 patients (165 intra-arterial therapy, 150 IV tPA controls) in the ESCAPE study [8], and 196 patients (98 intra-arterial therapy, 98 IV tPA controls) in the SWIFT PRIME study [9], all of which compared intra-arterial therapy to IV tPA alone. Unlike pharmacological therapy trials, interventional neuroradiology is characterized by several iterative steps in engineering- and experience-driven device development with tentative design improvements every few months. Once a randomized controlled trial is finished and published, the next device generation might be already available. Additionally, the benefit of other novel acute ischemic stroke treatment approaches, including neuroprotectant drugs [10] or hypothermia [11], also needs to be tested in randomized controlled trials.

However, the smaller the expected effect size of a new treatment approach compared to the current standard of care is, the more patients need to be recruited into fully powered randomized controlled trials. This is not only delaying patients’ access to promising new technology or treatments but also increases the costs for future medical devices or drugs. Furthermore, a considerable number of patients in a randomized controlled trial might be treated with a potentially less effective or even harmful treatment compared to current standard of care. To overcome these limitations, in silico evaluation of new treatment devices or drugs has gained increasing interest in some fields outside of acute ischemic stroke for efficacy testing [12,13].

**Figure 1:**
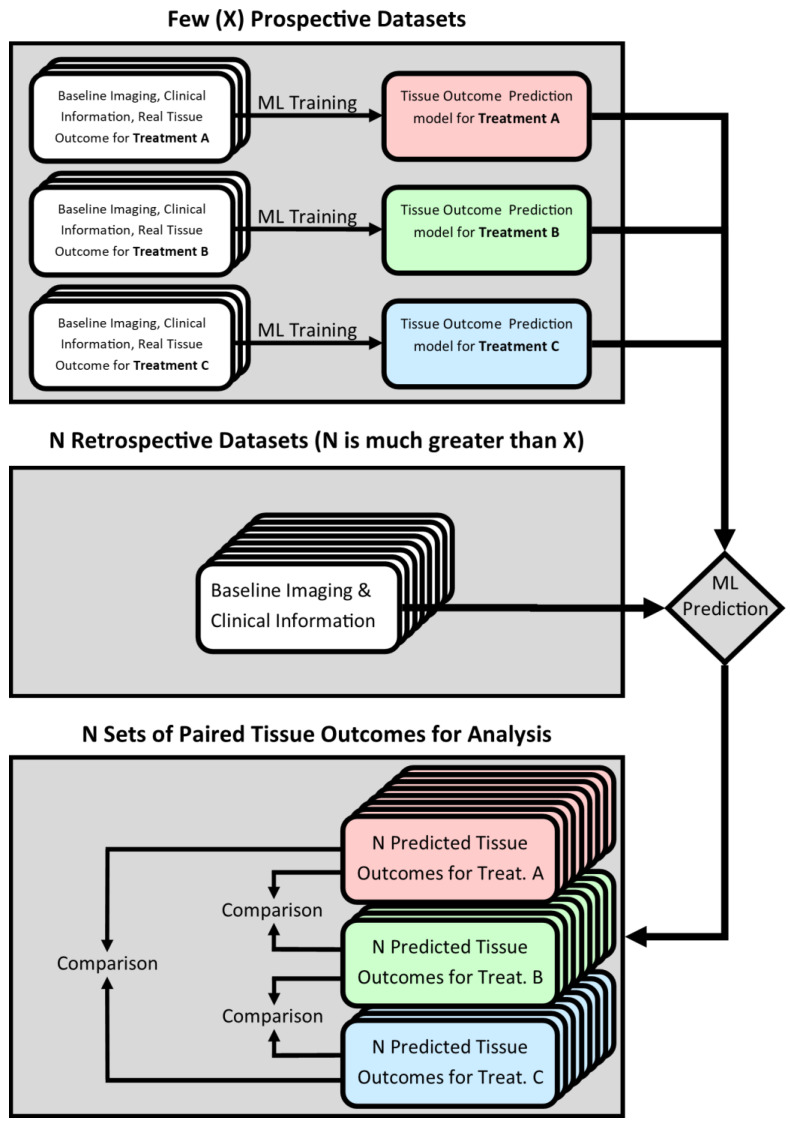
Graphical abstract illustrating the proposed in silico method.

The term “in silico” refers to the usage of computer methods for understanding biological processes in the living organism. In silico validation of medical devices is typically performed using a computational biomechanical model [14]. However, no such model exists for the evaluation of stroke treatments due to the high complexity of the cerebrovascular system and interventional procedure, which renders an in silico validation, for example using computational fluid dynamics simulations, infeasible. Instead, we propose the use of machine learning models, which have been used previously to model the evolution of acute ischemic stroke under particular treatment conditions [15,16,17,18,19,20,21,22,23]. In these studies, real patient data were used to empirically and automatically optimize (train) the machine learning model for the purpose of predicting patients’ final voxel-wise tissue outcome given their baseline imaging and clinical information. In case such a model is trained using data from patients treated exclusively with a particular drug or device, it can be assumed that it practically models the effect of the given treatment approach on the evolution of the ischemic lesion between the patients’ baseline and follow-up imaging. Thus, a collection of models, each trained to capture the effect of a different treatment, could be used to simulate hypothetical tissue outcomes for multiple treatment approaches in a single patient. Comparing patients’ predicted tissue outcomes between different treatment approaches would then provide a means of in silico efficacy testing for medical devices and drugs in the context of acute ischemic stroke. This study design is presented graphically in Figure 1.

Compared to previous randomized controlled trials, such an in silico approach could significantly reduce the burden of patient recruitment. For instance, treatment-specific models can be trained using a few datasets of patients treated with the methods to be compared. After training, those models can be applied to historical datasets that meet the trial’s inclusion criteria, i.e., the same criteria that were used to select the few patients for the actual treatment. While state-of-the-art deep machine learning approaches for tissue outcome prediction have been shown to lead to good results, they are generally believed to require a large sample size for training [24] so that these models are of reduced interest for the proposed in silico trial design. Compared to this, random decision forests (RDF) have been shown to have relatively small sample size requirements for tissue outcome prediction [25] while still producing competitive tissue outcome prediction results compared to other conventional machine learning techniques, such as generalized linear models and k-nearest neighbors [21]. However, the general framework proposed in this paper can be used with any machine learning model. In addition, the ability to compare treatment-contingent predicted tissue outcomes within, rather than between, patients could reduce the effects of inter-patient variability on the analysis, increasing its statistical power. Overall, introducing in silico evaluation methods to test new acute ischemic stroke treatment approaches is likely to increase the speed and power of these trials while decreasing the cost. The aim of this study is to evaluate if random decision forests for tissue outcome prediction can be used for the proposed in silico trial design, and whether they convey the aforementioned benefits.

### Article Contributions

The main contributions of this paper can be summarized as follows:

- An experimental framework is proposed for predicting the differential treatment effects of current gold-standard medical interventions using machine learning methods.
- The proposed in silico study setup is validated using a large database of real stroke patient data.
- It is demonstrated that treatment efficacy analysis using the proposed in silico framework benefits from an increase in statistical power.

## 2. Materials and Methods

### 2.1. Patient Data and Image Acquisition

The patient data used in this retrospective study, including information about image acquisition protocols and preprocessing methods utilized, have been described in detail in a previous publication [21]. These are summarized in the following for the reader’s ease of reference. Briefly described, this study utilized the anonymized data of 90 acute ischemic stroke patients with a major vessel occlusion of the ICA or MCA M1 segment treated at the University Medical Center Hamburg-Eppendorf, Germany. All patients used in this study were selected so that they fulfill typical guidelines for intra-arterial treatment. The patients were treated with an intravenous thrombolytic agent either exclusively (IV, *n* = 48) or in conjunction with intra-arterial mechanical thrombectomy (IA, *n* = 42). All study protocols and procedures were conducted in accordance with the ethical guidelines (Ethics committee of the Hamburg Chamber of Physicians, Hamburg, Germany) and in compliance with the Declaration of Helsinki. As this was a retrospective analysis, the requirement of informed consent was waived.

In all cases, the datasets available for each patient included diffusion-weighted (DWI) and perfusion-weighted (PWI) magnetic resonance imaging (MRI) acquired at admission, follow-up MRI or CT imaging acquired 5–7 days after stroke onset, and the following clinical variables: NIHSS score and age at admission, sex, time from symptom onset to imaging, and a binarized measure of recanalization status (TICI ≥ 2b). PWI and DWI datasets were acquired using a 1.5T Sonata or Avanto scanner (Siemens, Erlangen, Germany). Datasets were first divided by binarized recanalization status and then, for successfully recanalizing datasets, by treatment type to yield the following three patient groups: IA-recanalized (IAR, *n* = 33), IV-recanalized (IVR, *n* = 23), and non-recanalized (NR, *n* = 34). For this feasibility analysis, we used the non-recanalized patient group as an analogue for conservative treatment; recanalization status, being unavailable in the acute stage of stroke, is neither used for training nor prediction in the models.

### 2.2. Summary of Training Features

Tissue viability in acute ischemic stroke is a complex phenomenon that has a diverse set of contributing factors related to cerebral hemodynamics, patient-specific risks, differences in tissue vulnerability, and medical intervention. We selected features for model training with consideration for each of these factors as well as the design of prior tissue outcome prediction studies. Additional factors affecting stroke outcome are constantly being discovered [26]. However, for this study, we limited the data used for model training to that generally available for all patients in a retrospectively accessed database of clinical stroke patients.

#### 2.2.1. Voxel-Wise Medical Imaging Features

Biologically relevant hemodynamic changes in the human brain due to a stroke are typically observed and measured in the clinical setting using medical imaging techniques such as multi-modal MRI or CT to quantify their combined effects on a voxel-level scale. These voxel-level imaging parameters have historically been used to determine tissue viability using a threshold-based approach that segments the affected tissue into three compartments: (1) the ischemic core, which is already infarcted and cannot be salvaged, (2) the ischemic penumbra, which will eventually develop infarction without reperfusion, and (3) the benign oligemia, which is hypoperfused but not at risk of infarction [27]. These imaging parameters are considered sufficiently reliable that stroke treatment decisions are often based on the volume of the ischemic core and relative volume of the ischemic penumbra [28]. However, there is a growing acknowledgement that these imaging parameters are an oversimplification of the complex tissue perfusion and do not allow the making of true personalized outcome predictions [29].

Methods using machine learning to better interpret imaging parameters for stroke outcome prediction have already been proposed in a number of independent studies [15,16,17,18,19,20,21,22,23] and international challenges [30]. These methods typically consider the full range of imaging features previously used in clinical practice as salient predictors of tissue outcome. For perfusion-weighted imaging, these parameters are cerebral blood flow (CBF), cerebral blood volume (CBV), mean transit time (MTT), and time-to-maximum (Tmax). For diffusion-weighted MRI, this is the parameter apparent diffusion coefficient (ADC). All of these voxel-wise imaging parameters are included in this work in the machine learning model.

#### 2.2.2. Patient-Level Clinical Features

The extent to which imaging parameters predict poor tissue viability is likely mediated by several patient-specific factors. Infarction in the late hyperacute stage, for example, has been shown to depend not only on the magnitude of the hypoperfusion as indicated on imaging, but also the duration of the hypoperfusion [18]. Specifically, in patients who are rapidly recanalized, criteria based solely on imaging information may substantially overpredict the extent of the final infarct [31]. For this reason, we included the time from symptom onset to imaging as a training feature. Similarly, the mean values of perfusion and diffusion imaging parameters in healthy tissue are known to vary with respect to patient age [32,33] and sex [32]. Thus, both parameters are included as additional training features. Finally, we also included the NIHSS score at admission as it is a strong independent predictor of tissue fate in similar stroke outcome prediction models [17,18].

#### 2.2.3. Tissue-Specific and Spatial Features

Even within an individual brain, the infarction thresholds for perfusion and diffusion imaging parameters are known to vary between white and gray brain matter [34], between different brain regions [35] and, due to the spatial organization of stroke, likely with proximity to the ischemic core. Therefore, we included atlas-based white matter probabilities and brain region labels as training features. Because our model processes individual voxels without spatial context, we included the Euclidean distance of each voxel to the closest point in the ischemic core, as determined by ADC thresholding, as our final training feature.

#### 2.2.4. Medical Intervention

Although the treatment administered to a patient has a profound effect on their tissue outcome, we opted not to include the treatment as a training feature and to instead train a different model for each successful treatment of interest, as well as unsuccessful treatment as a surrogate for conservative treatment. The advantage of doing so is twofold. First, it enables the use of different training datasets for each model so that a model meant to learn one treatment effect does not learn from patients administered other treatments. Second, a model for a new treatment can be added to the ensemble or an existing model can be tweaked without having to retrain any of the other models.

### 2.3. Image Preprocessing and Feature Extraction

All imaging features described above were extracted for the patients available in this study using the AnToNIa software tool [36]. Briefly described, DWI acquired with diffusion weightings of b = 0 and b = 1000 s/mm^2^ were used to calculate a quantitative apparent diffusion coefficient (ADC) map, which was then used to segment the brain tissue, cerebrospinal fluid (ADC > 1200 × 10^−6^ mm²/s), and the ischemic core (ADC < 550 × 10^−6^ mm^2^/s). Subsequently, the ischemic core segmentation was used to compute a map of the shortest Euclidean distance from each voxel to the ischemic core. For each PWI dataset, maps of cerebral blood volume (CBV), cerebral blood flow (CBF), mean transit time (MTT), and time-to-maximum of the residual curve (Tmax) were calculated using a block-circulant singular value decomposition with a threshold of 0.15 [37]. The arterial input function needed for this was automatically identified using an atlas-based approach. For each follow-up image, an experienced medical expert used AnToNIa to manually segment the final lesion, yielding a binary mask of the real tissue outcome. Finally, image registration was used to transfer each perfusion parameter map (CBV, CBF, MTT, Tmax), the tissue type information (voxel-wise white and gray matter probabilities) and region labels from the MNI atlas, and the follow-up real tissue outcome to the patient’s ADC space. Clinical variables were associated with each voxel of the ADC image by simple repetition. As a result, each voxel was associated with a total of 12 features: ADC, distance to ischemic core, tissue type, anatomical location, CBV, MTT, Tmax, CBF, NIHSS, age, sex, and time from symptom onset, whereas the real tissue outcome was used as the outcome variable for training and testing of the machine learning models.

### 2.4. Machine Learning Model

Based on the preprocessed voxel-wise data, separate optimized random forest classifier models were trained to predict the voxel-wise treatment outcomes of successful IA therapy, successful IV therapy, and unsuccessful reperfusion therapy using datasets from the IAR, IVR, and NR groups, respectively. Training a separate prediction model for each treatment group, rather than a single combined model using the cohort information as a patient-level variable, guarantees that the effect of each treatment is learned independently of the other treatments being evaluated. In a combined model, for example, datasets from the IAR group would influence the prediction of treatment outcomes under IVR conditions, which could introduce significant bias.

Each classifier comprised 100 decision trees and was implemented using ALGLIB (www.alglib.net, accessed on 15 August 2021). For each classifier, the single hyperparameter *r*, representing the proportion of the training set used for the construction of individual trees, was set to 0.5. Stratified random undersampling was used to account for class imbalances resulting from the relatively small volume of the final infarct compared to the whole ipsilateral hemisphere. For each dataset, this entailed sampling every voxel that had a real infarct tissue outcome as well as an equal number of randomly selected non-infarct voxels from the ipsilateral brain parenchyma. A more granular description of this machine learning model has been previously published [21].

To virtually increase the sample size following the in silico principle described in the introduction, all three tissue outcomes (IAR, IVR, NR) were predicted for every patient available for this feasibility study. In all cases where the predicted treatment outcome matched the true treatment, which is referred to as ‘standard prediction’ in the following, the predicted and true tissue outcomes were compared using the Dice metric as a means of model validation. In all other cases, which are referred to as ‘cross-predictions’ in the following, the predicted tissue outcome represented an estimate of how the tissue outcome would look like if the patient had received the different treatment. In case of the standard predictions, a leave-one-out approach was used to maximize the quantity of datasets available. Essentially, each model was trained from scratch before every standard prediction using all datasets from the corresponding group except for the dataset for which the outcome was being predicted. For all cross-predictions, each model was trained only once using all data from one group without any omissions, since the dataset being predicted was not in the data partition used for training. For both the standard and cross-predictions, the predicted maps were postprocessed using morphological closing with a 3 × 3 × 3 kernel followed by connected-component analysis excluding components less than ten voxels in size as a means of noise reduction.

### 2.5. Statistical Evaluation

To test for significant differences between the predicted tissue outcomes simulating successful IA therapy, successful IV therapy, and unsuccessful reperfusion therapy, the predicted lesion volumes for all 90 patients were compared using a one-way repeated-measures ANOVA. In case of significant results, planned contrasts were performed between each pair of models using a Bonferroni-adjusted *p*-value to achieve a family-wise error rate of 5%. To demonstrate the difference between this analysis and one which does not use tissue outcome prediction models, a one-way ANOVA was conducted comparing the ground-truth lesion volumes between the corresponding patient groups (IAR, IVR, NR). A log transformation was applied to both the true and the predicted lesion volumes prior to conducting the respective ANOVAs to obtain variables that are normally distributed, as assessed by the Shapiro–Wilk test.

Confusion matrices and ROC analyses for the models were computed after applying the brain tissue masks derived from the ADC maps to exclude background and CSF voxels. Therefore, true positives were counted as the number of voxels positively labelled in all three of the brain tissue, predicted infarct, and ground-truth infarct segmentations. Conversely, true negatives were counted as the number of voxels positively labelled in the brain mask, but negatively labelled in both the predicted and ground-truth infarct segmentations. False positives and negatives were counted according to similar definitions, only considering voxels where the labels on the ground-truth infarct segmentation differed from the predicted infarct. Accuracy, sensitivity, specificity, and precision were derived from these four quantities according to standard definitions. Separate statistics were computed for each patient, then averaged together to avoid a heavy bias in the reported values toward larger lesions. AUC values were derived from the ROC curves using Simpson’s method.

The Dice metric, which is commonly used to quantify the overlap between two binary segmentations, is reported as a measure of the agreement between each model’s standard predictions and the corresponding ground-truth lesion segmentations. These Dice values were compared using a one-way ANOVA to determine if any model was significantly more reliable than another. Finally, the volume error of each predicted lesion was computed as a proportion of the true lesion volume and statistically compared between each pair of models using independent samples *t*-tests. All statistical analyses were performed in SPSS25.

## 3. Results

An in-depth description of the patient and imaging characteristics has been previously published [21]. Briefly summarized, there was no significant difference between any of the IAR, IVR, and NR patient groups with respect to the acute stroke lesion volume, penumbral tissue volume, follow-up lesion volume, age, sex distribution, or NIHSS scores at admission. However, the NR patients showed a significantly longer onset-to-imaging time compared to the IVR patients (*p* = 0.03).

**Figure 2:**
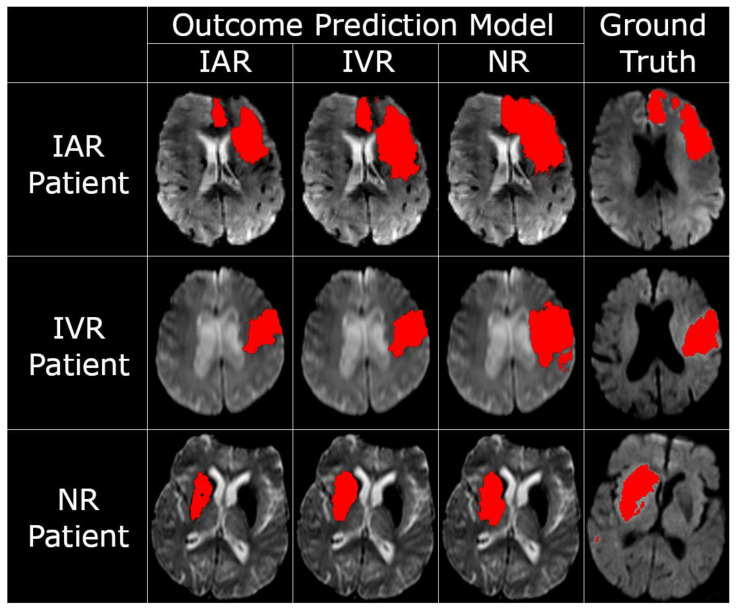
Selected follow-up lesion segmentations generated for each combination of outcome prediction model and patient, alongside the patients’ corresponding ground-truth follow-up lesion segmentation. Recanalization outcomes are abbreviated IAR (intra-arterial therapy, recanalizing), IVR (intravenous tPA, recanalizing), and NR (non-recanalizing).

The repeated-measures analysis, made possible by the proposed in silico trial design, revealed that the three sets of predicted lesion volumes were significantly different (*p* < 0.001). Planned contrasts indicated that the NR model predicted on average significantly larger lesion volumes (M = 68.75 mL, SD = 66.83 mL) compared to the IVR model (M = 49.67 mL, SD = 59.57 mL, *p* < 0.001), which in turn produced significantly larger lesion volumes than the IAR model (M = 34.42 mL, SD = 49.09, *p* < 0.001). Accordingly, the differences between the NR model and the IAR model (*p* < 0.001) were also significant. Exemplary tissue outcome predictions applying the three different models to the same patients are shown in Figure 2.

**Figure 3:**
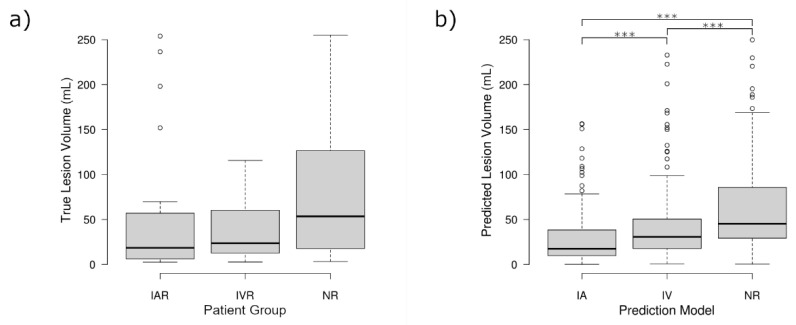
Boxplots representing the distribution of lesion volumes calculated for (**a**) the expert-segmented follow-up lesions, separated by the treatment outcome of the patient, (**b**) the predicted follow-up lesion, separated by predicted treatment outcomes. Recanalization outcomes are abbreviated IAR (intra-arterial therapy, recanalizing), IVR (intravenous tPA, recanalizing), and NR (non-recanalizing). Significance markers (***) represent *p* < 0.001 for post-hoc tests following the one-way ANOVA performed in (**a**) and the one-way repeated-measures ANOVA performed in (**b**). Circular markers represent outliers with values more than 1.5 times the interquartile range.

In contrast to the findings comparing the predicted lesion volumes, there was no significant difference comparing the real final lesion volumes between the three groups (*p* = 0.19). However, there was a non-significant trend between the three groups that followed the same pattern identified for the predicted lesion volumes, showing the smallest lesion volumes for IAR, slightly larger lesion volumes for IVR, and the largest lesion volumes for the NR group. The distribution of the real and predicted lesion volumes, and the similarities therein, can be seen in Figure 3.

The mean Dice values comparing the standard predictions to their corresponding ground-truths were not significantly different between the IAR, IVR, and NR machine learning models (*p* = 0.90). All three models produced mean Dice values > 0.44, which is comparable to similar tissue outcome prediction studies in the field of acute ischemic stroke [15,16,17,18,19,20,21,22,23]. Additionally, the proportional error, which quantifies the difference between the predicted and true lesion volume as a proportion of the true lesion volume, did not differ significantly between models (*p* = 0.60). These statistics can be found in Table 1.

**Figure 4:**
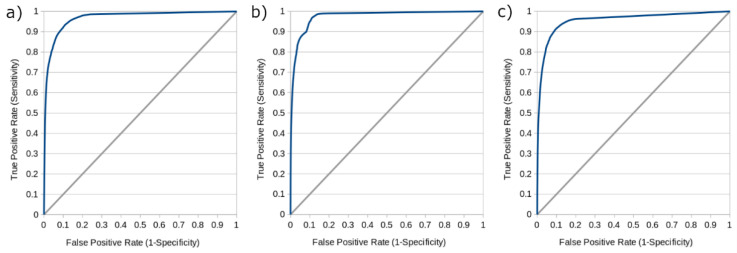
Receiver operating characteristic (ROC) curves for the standard predictions of each model. Recanalization outcomes for the models are (**a**) IAR (intra-arterial therapy, recanalizing), (**b**) IVR (intravenous tPA, recanalizing), and (**c**) NR (non-recanalizing). AUC values are (**a**) 0.979, (**b**) 0.976, and (**c**) 0.957.

Additional statistics including the mean accuracy, sensitivity, specificity, and precision of the models operating at their optimal thresholds are reported in Table 2, while Figure 4 shows receiver operating characteristic (ROC) curves derived from each model operating at thresholds varying from 0 to 1 by intervals of 0.01. The area under the ROC curve (AUC) for the IAR, IVR, and NR models were 0.979, 0.976, and 0.957, respectively. While these additional metrics are provided in keeping with the conventions of other machine learning studies in the field of stroke medicine [16,18,19,22,23,38], it should be noted that their interpretation is complicated by the fact that voxel-based tissue outcome prediction is a highly imbalanced problem. That is to say, the vast majority of brain tissue voxels will not proceed to infarction and are easy to identify as such, leading to especially high values for accuracy, specificity, and the area under the ROC curve (AUC), which may not intuitively reflect the ability of the model to discriminate between healthy and ischemic voxels.

## 4. Discussion

The main finding of this study was that the proposed in silico trial design leads to clinically feasible results, with successful recanalization following IA therapy resulting in significantly smaller predicted lesion volumes than successful recanalization following IV therapy, which in turn resulted in significantly smaller predicted lesion volumes than non-successful or conservative treatment. This trend is already well-known from previous studies comparing intra-arterial therapy [6] and intravenous thrombolysis [39], which confirms that the RDF models were able to train successfully on the relatively small dataset used for this study and make plausible treatment-specific tissue outcome predictions. By contrast, a direct comparison of the ground-truth lesion volumes for the three treatment outcomes yielded the same trend at a statistically insignificant level. This difference in statistical significance demonstrates that the proposed in silico trial design can be used to reduce the minimum sample size of new trials or provide early intermediate results that might be helpful to identify potentially unsuccessful trials at an earlier time point.

A major contributing factor to the significance of our results was likely the paired and repeated-measures statistical tests made possible by the in silico trial design, which offer several advantages over a conventional independent samples test. First, the paired and repeated-measures tests operate on each patient’s multiple predicted tissue outcomes, (as opposed to their single ground-truth tissue outcome), which represents a virtual expansion of the study sample size. Second, these tests compare the multiple predicted tissue outcomes within, rather than between, individual patients, which serves to control for the between-subjects variability and further increase the statistical power of the analysis.

It should also be noted that all patients used in this study were selected so that they fulfilled typical guidelines for intra-arterial treatment. The patients in the NR group only showed a significantly longer symptom onset-to-imaging time than patients in the IVR group while all other clinical characteristics were similar. This suggests that the ability to predict treatment effects was learned by the machine learning models and not simply inherited from bias in the training or testing data. The absence of significant differences in the proportional volume error of the models further suggests that the results are not biased by a systematic underprediction of lesion volumes by the IAR model nor an overprediction of lesion volumes by the NR model. The quality of the standard predictions from the three models, as evaluated by the Dice coefficient, was also found to be not significantly different. Taken together, it is reasonable to conclude that the random forest models were successfully able to learn the differential treatment effects of successful IA therapy, successful IV therapy, and unsuccessful therapy used as a proxy for conservative treatment. Within this context, it should be noted that a much higher reperfusion/recanalization rate is typically observed for IAR compared to IVR treatment. Since the IAR and IVR treatment machine learning models developed in this work both assume successful treatment in all cases (as only those patients were used for training), the effects identified are rather an underestimation of the true effects that would be found when comparing the two clinical treatment cohorts without taking the recanalization success into account, which gives even more credibility to the ability of the suggested in silico modeling approach to being able to determine significant treatment effects even with a small sample.

The proposed in silico evaluation scheme might be desirable for research and clinical studies in general, where patient recruitment can be costly and labor-intensive, but is especially relevant in situations where it is difficult to recruit a large number of patients or to match patients between groups. For example, clinical trials are warranted to examine the prospective benefit of mechanical thrombectomy in less-common patient demographics such as those with occlusion of the posterior circulation [40,41], as well as children and adolescents [42,43]. For these demographics in particular, the limited availability of patient datasets may hinder efforts to construct a sufficiently powered regular study. The proposed method would allow researchers to acquire only a few datasets for model training, then apply these trained models to the large number of historical datasets available in most primary stroke care centers for the in silico evaluation. In silico outcome prediction may also have clinical utility in the field of computer-aided decision making. For example, comparing the predicted lesions for new stroke patients under different treatment conditions could assist clinicians in determining the most appropriate treatment on an individual basis, though this idea remains to be evaluated in future studies.

It should be mentioned that this is not the first study to use predictive modeling approaches for an in silico evaluation [44]. Fiehler et al. used a similar machine learning model based on CTP datasets to evaluate the effectiveness of a new recanalization device together with a specific intermediate catheter compared to intravenous tPA [15]. The major difference is that, in the cited clinical study, only a single model was trained (using data from patients treated with tPA). This trained machine learning model was then applied to the patients treated with the new recanalization device and the volume of saved tissue was quantified by comparing the true lesion volume in these patients with the predicted tPA treatment lesion volume. Compared to this, the proposed method of training multiple machine learning models has several theoretical benefits. First, one can make full use of many historical datasets available for the actual in silico evaluation as the real lesion outcome is not required. Second, it is statistically more sound to compare outcomes that were generated using the same method. Comparing a true lesion outcome with the predicted lesion outcome might be biased, for example, due to regularization used within the machine learning models. Additionally, blood clots can move downstream or break up into smaller clots because of the treatment so that the anatomical lesion location varies between baseline and follow-up imaging, which cannot be accounted for when comparing true and virtual lesion outcomes. While this is less of a problem for the direct comparison of two lesion predictions, it would be important to exclude these cases from the model training.

There is also a much larger body of knowledge regarding predictive modeling for stroke outcome prediction outside the context of in silico treatment evaluation. Many of the earliest machine learning models trained for stroke tissue outcome prediction were based on linear [17,22] or logistic [18] regression. While these methods were useful for quantifying the contribution of particular variables to the overall infarct probabilities [17], more recent publications comparing regression models and decision forests unanimously agree that decision forests facilitate more accurate tissue outcome predictions [16,19,21]. This was the motivating factor behind using decision forests in the current work. However, it should be noted that the purpose of this investigation was not to propose the next state-of-the-art model for tissue outcome prediction, but rather to propose an in silico framework for treatment evaluation that can be used with any tissue outcome prediction model. More recently, there are more works using deep learning models to predict stroke tissue outcomes from imaging and clinical data showing high accuracy [23]. Following trends in other medical domains [24], it seems likely that deep learning models will significantly outperform conventional machine learning models for stroke tissue outcome prediction. However, this remains to be shown via comparative studies. In this context, the results of the current study have a couple of important implications. First, we show that deep learning is not strictly necessary for the proposed in silico approach. This may be especially significant for studies where data are scarce or difficult to obtain, considering the relatively small sample size requirements of decision forests [25]. Conversely, deep learning methods are uniquely suited to leverage the large amount of data present in electronic health records, otherwise known as clinical big data [45]. Previous predictive modeling studies have used a few additional variables including age [15,17], sex [15,17], admission NIHSS [15,17,18], and tissue priors [16,17] to enrich the training data of their stroke tissue outcome models. The current study includes all of these as training features. However, if more salient variables are discovered, it may become less feasible to use conventional (non-deep) classifiers. In this eventuality, the proposed in silico method could still be used alongside deep learning classifiers to take advantage of big medical data. Second, we provide a benchmark method for the evaluation of future deep learning classifiers. Demonstrating the prospective benefit of deep learning in the context of in silico treatment evaluation requires a standardized, accessible, and well-validated comparison method. Random forests are already implemented in a number of freely available software packages and have been repeatedly validated as high-performing stroke tissue outcomes in the existing literature [15,16,19,21]. As novel deep learning models (and other future methods) are explored as a way to improve the performance of the proposed in silico method, we expect this study to provide a valuable point of reference.

There are some limitations of this work that have to be mentioned. First, large clinical stroke trials typically use patients’ disability and functional independence score on the modified Rankin Scale (mRS) as the primary endpoint because the mRS can be evaluated consistently across trials and conveys no additional risk to the patient [46]. In comparison, the current study analyzes patients’ follow-up lesion volume, which is a common secondary endpoint in clinical stroke trials. However, there is a strong inverse correlation between the likelihood of a good functional outcome (mRS 0–2) and the volume of the patient’s follow-up lesion volume [47,48]. It has been reported that this correlation also holds true for follow-up lesions predicted from acute PWI data [49], raising the possibility that mRS scores could be predicted directly from acute image data and clinical information in future and used in the same manner as the prediction lesion volume for an in silico evaluation. As mRS is used as both a clinical and a functional outcome, an imaging-based approach to predicting mRS would likely benefit from the availability of information about the infarct location, which adds valuable context when interpreting the functional severity of stroke from the lesion volume [50], as well as the potential to add salient covariates [51] and additional neurological data [38] to the model. More recently, it has been suggested that the post-treatment NIHSS score may be a suitable alternative primary outcome measure to mRS for clinical trials of acute ischemic stroke [52]. One compelling argument for the incorporation of post-treatment NIHSS into future predictive models is that especially high or low post-treatment NIHSS scores are associated with discrepancies between patients’ post-treatment infarct volume and 90-day mRS [53]. Beyond using post-treatment NIHSS as a surrogate for mRS, there is the potential to use the true or predicted post-treatment NIHSS alongside the voxel-wise tissue fate as each appears to provide context for the other in determining the patient’s true functional outcome. Generally, it would be possible to include any kind of clinical or functional outcome score into the framework as long as it can be predicted with reasonable accuracy from the baseline imaging data or predicted lesion outcome.

A second limitation of this study is that it employed a leave-one-out cross-validation approach to a single-center dataset, making a full discussion of the study’s external validity difficult. Within this context, it should be noted that machine learning models that were trained using datasets from a single institution should not be applied to a multi-center database of historical datasets without caution, as differences due to image acquisition or patient selection might bias the machine learning models. In this case, it would be more appropriate to also train the machine learning models using datasets from the same centers where the test datasets were acquired. More generally, care must be taken to ensure that the training data for a model is representative of the data that it is applied to. An insufficient quantity of training data or the presence of significant groupwise differences between the training and test datasets could bias the results of an in silico experiment. Investigating a novel treatment device, patient demographic, or complication, for example, would require that a new model be trained using a sample of the novel data. Finally, it should be noted that the proposed framework still needs be validated in more detail using prospectively collected trial data, such as a multi-center, double-blind, randomized controlled ESCAPE-NA1 trial [54] or other recent studies.

## 5. Conclusions

The results of this study suggest that machine learning models can be used for an efficient in silico trial design that leads to clinically plausible results and could be used for evaluating the efficacy of new treatment devices or approaches for acute ischemic stroke patients. Even though the proposed in silico study design has the potential to significantly accelerate acute stroke studies and reduce sample sizes, the proposed method should be rather considered as a valuable tool to support clinical trials (e.g., early intermediate efficacy analyses) rather than replace them entirely at this point as this approach still needs to be evaluated and validated in more detail.

## Figures and Tables

**Table 1:** Descriptive statistics comparing each model’s standard predictions and the corresponding follow-up lesion segmentations. Recanalization outcomes are abbreviated IAR (intra-arterial therapy, recanalizing), IVR (intravenous tPA, recanalizing), and NR (non-recanalizing).

	Dice Coefficient		Proportional Volume Error	
	Mean	Standard Dev.	Median	Interquartile Range
IAR Model	0.442	0.231	0.182	1.80
IVR Model	0.448	0.255	−0.139	2.10
NR Model	0.473	0.245	0.054	1.59

**Table 2:** Additional statistics derived from the confusion matrices comparing each model’s standard predictions and the corresponding follow-up lesion segmentations. Recanalization outcomes are abbreviated IAR (intra-arterial therapy, recanalizing), IVR (intravenous tPA, recanalizing), and NR (non-recanalizing).

	Accuracy		Sensitivity		Specificity		Precision	
	Mean	Std. Dev.	Mean	Std. Dev.	Mean	Std. Dev.	Mean	Std. Dev.
IAR Model	0.978	0.026	0.599	0.276	0.990	0.013	0.507	0.308
IVR Model	0.986	0.009	0.567	0.263	0.993	0.007	0.474	0.298
NR Model	0.971	0.022	0.619	0.295	0.984	0.017	0.489	0.279

## Data Availability

The datasets generated during and/or analyzed during the current study are not publicly available due to containing information that could compromise the privacy of research participants but are available from the corresponding author on reasonable request.
